# The impact of having both cancer and diabetes on patient-reported outcomes: a systematic review and directions for future research

**DOI:** 10.1007/s11764-015-0486-3

**Published:** 2015-10-01

**Authors:** Pauline A. J. Vissers, Louise Falzon, Lonneke V. van de Poll-Franse, Frans Pouwer, Melissa S. Y. Thong

**Affiliations:** CoRPS—Center of Research on Psychology in Somatic diseases, Department of Medical and Clinical Psychology, Tilburg University, P.O. Box 90153, 5000 LE Tilburg, The Netherlands; Department of Research, Netherlands Comprehensive Cancer Organisation, Eindhoven, The Netherlands; Center for Behavioral Cardiovascular Health, Columbia University Medical Center, New York, NY USA

**Keywords:** Cancer, Diabetes, Patient-reported outcomes, Systematic review, Health-related quality of life

## Abstract

**Purpose:**

This systematic review aims to summarize the current literature regarding potential effects of having both cancer and diabetes on patient-reported outcomes (PROs) and to provide directions for future research.

**Methods:**

MEDLINE, The Cochrane Library, CINAHL, and PsycINFO were searched from inception to January 2015. All English peer-reviewed studies that included patients with both cancer and diabetes and assessed PROs were included. All included studies were independently assessed on methodological quality by two investigators.

**Results:**

Of the 3553 identified studies, 10 studies were included and all were considered of high (40 %) or adequate (60 %) methodological quality. Eight of the 10 studies focused on health-related quality of life (HRQoL), functioning, or symptoms and 2 studies assessed diabetes self-management. Overall, HRQoL and functioning was lower, and symptoms were higher among patients with both cancer and diabetes as compared to having cancer or diabetes alone. Furthermore, one study reported that diabetes self-management was impaired after chemotherapy.

**Conclusions:**

Having both cancer and diabetes resulted in worse PROs compared to having either one of the diseases, however, the considerable heterogeneity of the included studies hampered strong conclusions. Future studies are needed as this research area is largely neglected. As the majority of the included studies focused on HRQoL, future research should address the impact of both diseases on other PROs such as depression, patient empowerment and self-management.

**Implications for Cancer Survivor:**

Having both cancer and diabetes might result in worse PROs, however, more research is needed as current evidence is scarce.

**Electronic supplementary material:**

The online version of this article (doi:10.1007/s11764-015-0486-3) contains supplementary material, which is available to authorized users.

## Introduction

Due to the increased aging of the population, early detection, and better treatment of diseases, the number of cancer survivors is increasing [1]. As a result, more and more cancer survivors live with other chronic diseases of which diabetes is one of the most prevalent [2]. The prevalence of concurrent diabetes among cancer patients depends on cancer type, gender, and age at diagnosis and varies from 8 % among prostate cancer patients to approximately 26 % among pancreas cancer patients aged 65 years or older [2]. This high prevalence of diabetes among cancer patients results in worse outcomes and increases the burden on health systems worldwide.

The link between cancer and diabetes is extensively studied in recent literature and is mainly focused on the impact of diabetes on cancer incidence and mortality. Recent meta-analyses show that diabetes is strongly associated with the development of pancreatic (OR = 1.82, 95 % CI: 1.66–1.89) [3], liver (OR = 2.50, 95 % CI: 1.80–3.50) [4], and endometrial cancer (RR = 2.10, 95 % CI: 1.75–2.53) [5]. Moderate, positive associations have been reported for diabetes and breast (RR = 1.20, 95 % CI: 1.12–1.28) [6], colorectal (RR = 1.26, 95 % CI: 1.05–1.50) [7], and bladder (RR = 1.24, 95 % CI: 1.08–1.42) [8] cancer incidence, while diabetes has been associated with a decreased incidence of prostate cancer (RR = 0.84, 95 % CI: 0.76–0.93) [9]. Furthermore, previous research shows that having diabetes is associated with a 30–40 % increased mortality risk among cancer patients, which was mainly apparent among breast, endometrial, and colorectal cancer patients [10, 11].

As the group of patients with both cancer and diabetes is growing, patients’ experience of living with both diseases is becoming more important. However, this research area is largely neglected. Patient-reported outcome (PRO) assessments such as health-related quality of life (HRQoL), functioning, and symptoms are needed as it is plausible that patients with multiple chronic diseases experience more problems. This knowledge is essential to improve clinical practice and care for this growing group of patients.

A significant number of cancer survivors consistently report lower physical functioning, sexual functioning, and more symptoms of distress and fatigue [12, 13]. Similarly, diabetes patients are more likely to suffer from depression [14], report a lower quality of life [15], and lower sexual functioning [16]. As both cancer and diabetes patients report deteriorated PROs compared to people without the disease, we hypothesize that having both chronic diseases will result in even more deteriorated PROs. The aim of this systematic literature review is to summarize the current knowledge on the impact of having both cancer and diabetes on PROs. In addition, as we expect that this research area will be largely neglected, we also aim to provide directions for future research.

## Methods

### Search strategy

LF conducted the systematic literature search on August 2013 and updated the search on January 2015. The following databases were included: MEDLINE, The Cochrane Library, CINAHL, and PsycINFO. Subject headings and freetext terms for diabetes (i.e., diabet* OR diabetes mellitus) were combined with search terms for cancer (i.e., cancer* OR neoplasm* OR oncolog*). As PROs cover a wide range of different aspects, we did not include any search terms for PROs to avoid missing relevant papers. The full search strategy is shown in Online Resource 1. After the search was conducted, the cited references of the selected studies were searched using Web of Science and their references lists checked; in addition, PubMed related articles were used for the two most recent included studies to identify studies that were not found with the initial literature search.

### Selection criteria

All retrieved studies (including abstracts of unpublished studies) were screened and studies that met the following four selection criteria were included: (1) the study is focused on patients with both cancer and diabetes, (2) PRO is primary or secondary outcome measure of the study, (3) is published in a peer-reviewed journal, and (4) is published in English. Studies that assessed the effects of several chronic or comorbid diseases, including diabetes, among cancer patients on PROs were not included as the studies should have a primary focus on both cancer and diabetes. Similarly, studies that aimed to address comorbid or chronic diseases, including cancer, among diabetes patients were excluded.

### Quality assessment

Each selected study was independently scored on methodological quality by 2 reviewers (PV and MT) based on a set of 14 quality criteria (Table 1). These quality criteria were based on established criteria lists used in previous studies [17, 18]. Disagreements between the reviewers on the quality criteria were resolved during a consensus meeting. All studies received 1 point for each of the 14 quality criteria that was met. If a criterion was not met or described insufficiently, 0 point was assigned. Thus, each study can obtain a maximum score of 14 points. Studies that scored 75 % or more of the maximum attainable score (i.e., ≥11 points) were considered as “high quality study,” studies scoring between 50–75 % (i.e., 7–10 points) were considered of “adequate quality,” while those scoring <50 % (i.e., ≤6 points) were considered of “low quality.” These criteria were arbitrarily chosen and based on previous research [17].

**Table 1:** List of criteria for assessing the methodological quality of studies on patient-reported outcomes among patients with cancer and diabetes

Positive if with respect to	Number of studies that scored positive
Patient-reported outcomes	*N* (%)
1. Examining PROs was a primary objective of the study	10 (100)
2. A validated questionnaire to measure PROs was used	10 (100)
Study population	
3. The patient sampling process is described	10 (100)
4. A (healthy) normative sample is included for comparison	3 (30)
5. Patients with both cancer and diabetes are compared to either patients with only cancer or only diabetes on at least two sociodemographic variables	8 (80)
6. A description is included of at least two clinical variables regarding cancer diagnosis (e.g., cancer stage, treatment, time since cancer diagnosis)	8 (80)
7. A description is included of at least two clinical variables regarding diabetes diagnosis or severity (e.g., HbA_1c_ levels, treatment, time since diabetes diagnosis)	3 (30)
8. Inclusion and/or exclusion criteria are described	9 (90)
9. Participation rates for patient groups are described and these are >75 %	4 (40)
10. Information is given regarding differences in demographic and/or clinical characteristics of respondents vs non-respondents	3 (30)
Study design	
11. The study sample includes at least 75 patients (arbitrarily chosen)	8 (80)
12. The process of data collection is described	8 (80)
13. The difference in the outcome variable between cancer patients with diabetes and patients with only cancer and/or only diabetes is assessed in multivariable models, including at least 2 covariates	8 (80)
Results	
14. Mean, median, standard deviations, or percentages are reported and compared between cancer patients with diabetes and patients with only cancer and/or only diabetes for the most important outcome measures	8 (80)

## Results

### Description of the included studies

**Fig. 1:**
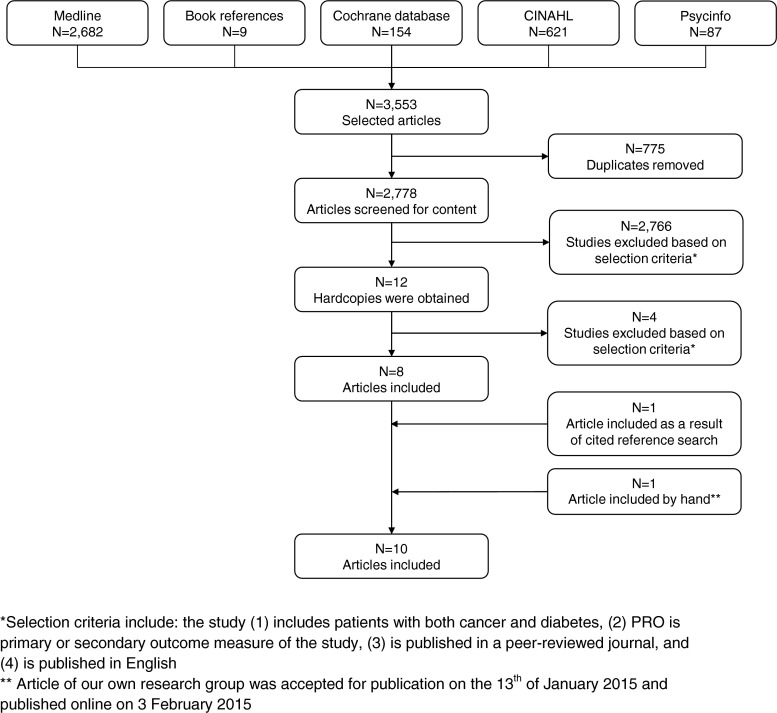
Flow chart of the selection process of the systematic literature search

The initial broad search strategy on cancer and diabetes that did not include a term for “PROs” yielded 3553 hits, and after the removal of duplicates and the application of selection criteria, a total of 10 studies were included in this study, of which 2 were based on the same data [19, 20] (Fig. 1). Eight of the included studies had a sample size of at least 590 participants, while 2 studies, based on the same data, included 43 patients [19, 20] (Table 2). The number of patients with both cancer and diabetes was rather low; 5 studies included less than 100 patients with both diseases [19–23]. Moreover, only 4, of which 3 unique, studies had a longitudinal design [19, 20, 24, 25], while the other 6 studies addressed the associations between cancer and diabetes and PROs cross-sectionally [21–23, 26–28]. Most studies focused on patients with specific cancer types including patients with diabetes and prostate [22–25], colorectal [27, 28], or breast cancer [23]. Five studies included cancer patients with diabetes (CA + DM+) and made a comparison with cancer patients without diabetes (CA + DM-) [21, 22, 24, 25, 28], one study compared CA + DM+ patients with patients with diabetes only (CA-DM+) [23] and two studies included CA + DM-, CA-DM+ and patients without both diseases (CA-DM-) for comparison [26, 27]. Two studies, based on the same data, only included CA + DM+ and did not include a comparison group [19, 20]. Of the 10 included studies, 8 focused on HRQoL, self-perceived health status, functioning, or symptoms, while 2 studies assessed the impact of cancer and its treatment on diabetes self-management. Most studies used a validated questionnaire. The Short Form (SF)-36 was used most frequently to assess HRQoL or self-perceived health status [21, 22, 25], other studies used the Health Utility Index Mark 3 (HUI3) [26], the EuroQoL Group’s EQ-5D [23], the Audit of Diabetes Dependent Quality of Life (ADDQoL) [23], the European Organization for Research and Treatment of Cancer core Quality of Life Questionnaire (EORTC QLQ-C30) [27], or the University of California, Los Angeles, Prostate Cancer Index (UCLA-PCI) [22, 24, 25]. The EORTC QLQ-Chemotherapy-Induced Peripheral Neuropathy (CIPN)-20 was used to assess neuropathic symptoms [28]. Eight out of 10 studies conducted multivariate analyses and mainly adjusted for socio-demographic [19, 21, 22, 24–28] and cancer-related covariates [19, 21, 22, 25, 27, 28], while diabetes-related covariates [19] and lifestyle factors [24, 26–28] were less often adjusted for.

**Table 2:** Overview of the included studies

Study	Country	Design	Study sample		Instrument	Results	Quality score
Health-related quality of life/self-perceived health/functioning							
Bowker et al. (2006)	Canada	Cross-sectional	113,587 patients with or without cancer (any type)	CA + DM+: 207	HUI3	HUI3 score:	10
				CA + DM-: 1692		CA + DM+ vs CA-DM-: *b* = −0.10 (95 % CI: −0.13 to −0.09), *P* value <0.0001	
				CA-DM+: 4394		CA-DM+ vs CA-DM-: *b* = −0.04 (95 % CI: −0.05 to −0.04), *P* value <0.0001	
				CA-DM-: 107,295			
						CA + DM- vs CA-DM-: *b* = −0.04 (95 % CI: −0.05 to −0.03), *P* value <0.0001	
Hershey et al. (2012a)	USA	Cross-sectional	661 patients with cancer (any type)	CA + DM+: 76	SF-36	Physical functioning:	9
				CA + DM-: 585		CA + DM- vs CA + DM+: *b* = 12 (95 % CI: 7–18), *P* value <0.0001	
Latini et al. (2006)	USA	Longitudinal	1248 prostate cancer patients	CA + DM+: 117	UCLA-PCI	Urinary function at follow-up:	10
				CA + DM-: 1131		CA + DM+ vs CA + DM-: 72 ± 24 vs 77 ± 22, *P* value = 0.01	
Mols et al. (2008)	The Netherlands	Cross-sectional	590 prostate cancer patients	CA + DM+: 65	SF-36	General health:	13
				CA + DM-: 525	UCLA-EPCI	CA + DM+ vs CA + DM-: *b* = −0.13, *P* value <0.01	
						Vitality:	
						CA + DM+ vs CA + DM-: *b* = −0.12, *P* value <0.01	
Onitilo et al. (2013)	Australia	Cross-sectional	3466 diabetes patients either with or without a history of breast or prostate cancer	Breast cancer:	EQ-5D	In men with prostate cancer only:	11
				CA + DM+: 77	ADDQoL	Problems with mobility:	
				CA-DM+: 1470		CA + DM+ vs CA-DM+: 51 % vs 29 %, *P* value <0.001	
				Prostate cancer:		Problems in usual activities:	
				CA + DM+: 81		CA + DM+ vs CA-DM+: 35 % vs 25 %, *P* value = 0.035	
				CA-DM+: 1838			
Thong et al. (2011)	USA	Longitudinal	1811 prostate cancer patients	CA+ and incident DM: 215	SF-36	At baseline those with prevalent diabetes report significant lower HRQoL, but after adjustments in longitudinal analyses no differences in HRQoL between CA + DM+ and CA + DM- were observed.	10
				CA+ and prevalent DM: 239	Individual items on BF and SF		
				CA + DM-: 1357			
Vissers et al. (2014)	The Netherlands	Cross-sectional	2761 patients with or without colorectal cancer and/or diabetes	CA + DM+: 328	EORTC-QLQ-C30	Physical functioning	12
				CA + DM-: 1731		CA + DM+ vs CA + DM-: beta = −3.8, *P* value <0.01	
				CA-DM+: 78		Male sexual problems	
				CA-DM-: 624		CA + DM+ vs CA + DM-: beta = 9.4, *P* value <0.01	
Vissers et al. (2015)	The Netherlands	Cross-sectional	1193 colorectal cancer patients	CA + DM+: 218	EORTC-QLQ-CIPN20	Neuropathic symptoms—tingling fingers or hands	11
				CA + DM-: 975		CA + DM+ vs CA + DM-: OR = 1.40 (95 % CI: 1.00–1.94)	
						Neuropathic symptoms—tingling toes or feet	
						CA + DM+ vs CA + DM-: OR = 1.47 (95 % CI: 1.04–2.07)	
						Neuropathic symptoms—numbness in toes or feet	
						CA + DM+ vs CA + DM-: OR = 1.83 (95 % CI: 1.28–2.62)	
						Neuropathic symptoms—males; erection problems	
						CA + DM+ vs CA + DM-: OR = 1.83 (95 % CI: 1.11–3.03)	
Diabetes self-management							
Hershey et al. (2012b)	USA	Longitudinal	43 patients with a solid tumor and type I or II DM		SIC, modified intrusiveness of illness inventory, SCI-R	Lower diabetes self-management after 8 weeks on chemotherapy as compared to baseline: 45.86 ± 2.65 vs. 50.84 ± 2.47	7
						Higher symptom burden after 8 weeks on chemotherapy as compared to baseline: 32.57 ± 4.49 vs. 25.43 ± 3.81	
						Overall impact on diabetes self-management was moderate (16.47 ± 8.43), highest impact on: exercise (4.35 ± 2.36), blood sugar monitoring (3.73 ± 2.38) and ability to eat and drink (3.56 ± 2.31)	
						Positive correlation between impact of cancer on diabetes self-management and symptom burden at 8 weeks (*r* = 0.46, *P* = 0.004)	
Hershey et al. (2014)	USA	Longitudinal	43 patients with a solid tumor and type I or II DM		SIC, DCI, CIDS, OE, HADS, SCI-R	Living arrangements, years with DM, total number of medications, baseline DM self-management, DM self-efficacy and baseline and 8-week symptom severity were significant predictors of diabetes self-management.	7

*ADDQoL* Audit of Diabetes Dependent Quality of Life, *CIDS* Confidence In Diabetes Self-care, *DCI* Diabetes Complication Index, *EORTC QLQ*-*C30* European Organization for Research and Treatment of Cancer core Quality of Life Questionnaire, *EPIC* Expanded Prostate Cancer Index Composite, *EQ*-*5D* EuroQol Group’s EQ-5D, *FLIC* Functional Living Index Cancer, *HADS* Hospital Anxiety and Depression Scale, *HUI3* Health Utility Index Mark 3, *OE* outcome expectancies, *SCI*-*R* Self-Care Inventory Revised, *SIC* Symptoms of Illness Checklist, *SF-36* Short Form 36, *UCLA*-*PCI* University of California, Los Angeles, Prostate Cancer Index

### Study quality

The 10 included studies scored a mean quality score of 10 out of 14, and scores ranged between 7 and 13. Four studies (40 %) were classified as being of high quality and 6 (60 %) of adequate quality according to our quality criteria. No studies were considered of low quality. The criteria that were least often met are (#4) the inclusion of a (healthy) normative sample for comparison, (#7) a description of at least two clinical variables regarding diabetes diagnosis, and (#10) information is given regarding differences in demographic and/or clinical characteristics of respondents vs non-respondents (all met by 3 studies) (Table 1).

### HRQoL, functioning, and symptoms

All included studies reported worse PROs among CA + DM+ compared to CA + DM-, CA-DM+, or CA-DM- on at least 1 studied item or subscale, except for 1 longitudinal study [25]. Nine out of the 10 included studies assessed more than 1 PRO, while 1 study only included a general measure of HRQoL [26].

### General HRQoL

A large cross-sectional study conducted in Canada reported lowest average HRQoL scores for CA + DM+ (*n* = 940) followed by CA + DM- (*n* = 1,692), CA-DM+ (*n* = 4,394), and CA-DM- (*n* = 107,295) patients with average HUI3 scores ranging between 0.67 and 0.89 (i.e., where −0.36 = worst possible health, 0 = death, and 1 = perfect health) [26]. The HUI3 indirectly measures HRQoL using 8 attributes (vision, hearing, speech, ambulation, dexterity, emotion, cognition, and pain) and a mean difference of 0.03 was considered as clinically important. Multivariable regression analyses showed similar results with a lower HRQoL for CA + DM+, CA + DM-, and CA-DM+ patients as compared to CA-DM- patients with beta’s of −0.10, −0.04, and −0.04, respectively, which was regarded clinically relevant [26]. Similarly, lower general health was reported in a cross-sectional study among 65 prostate cancer patients with vs 525 without diabetes with average SF-36 scores of 51.9 vs 62.5, which remained significant in multivariable analyses (beta = −0.13) [22]. A longitudinal study among prostate cancer patients did observe differences between CA + DM+ and CA + DM- in general health at baseline, but after adjustments for age, marital status, educational level, income, employment status, baseline HRQoL, cancer stage, primary treatment, baseline PSA, and baseline Gleason score, this difference did not remain significant [25]. Other studies did not report a worse general health among those with both cancer and diabetes [23, 27].

### Physical functioning or mobility

Five studies included a measure of physical functioning or mobility. In a study with 76 CA + DM+ and 585 CA + DM-, CA + DM+ scored on average 12 points lower on the physical functioning subscale of the SF-36 as compared to CA + DM- [21], as this difference was larger than 0.5 times the standard deviation it can be considered to be clinically relevant [29]. Similarly, a cross-sectional study found more problems with mobility and usual activities among men with prostate CA + DM+ as compared to CA-DM+, but this difference was not found among women with breast cancer [23]. Colorectal CA + DM+ reported a worse physical functioning as compared to CA + DM- (beta = −3.8) [27]. Two studies did not report lower physical functioning among CA + DM+ [22, 25], however, one study did report lower vitality among prostate CA + DM+ as compared to CA + DM- (beta = −0.12), which was considered a clinically relevant difference [22].

### Sexual functioning

Sexual functioning was assessed in one study among colorectal CA + DM+ [27] and in two studies with prostate CA + DM+ [24, 25]. Colorectal CA + DM+ reported more male sexual problems compared to colorectal CA + DM- (beta = 9.4) in a cross-sectional study from the Netherlands [27]. Among prostate cancer patients, two longitudinal studies did not observe a significant association between comorbid diabetes and sexual functioning [24, 25].

### Urinary and bowel functioning

Three studies among prostate cancer CA + DM+ and CA + DM- patients also focused on prostate cancer-specific symptoms, including urinary functioning and/or bowel functioning [22, 24, 25]. One study reported lower urinary function during follow-up among prostate CA + DM+ as compared to CA + DM- (mean score 72 ± 24 vs 77 ± 22) [24], but the other studies did not report differences in urinary or bowel functioning [22, 25].

### Neuropathic symptoms

A cross-sectional study by our research group among 218 colorectal CA + DM+ and an age- and sex-matched sample of 975 CA + DM- patients assessed differences in neuropathic symptoms. CA + DM+ patients reported more neuropathic symptoms regardless of cancer treatment as compared with CA + DM- patients regarding tingling fingers or hands (OR = 1.40; 95 % CI: 1.00–1.94), tingling toes or feet (OR = 1.47 95 % CI: 1.04–2.07), numbness in toes or feet (OR = 1.83; 95 % CI: 1.28–2.62), and erection problems among men (OR = 1.83; 95 % CI: 1.11–3.03) [28]. However, the majority of reported symptoms were of mild severity.

### Mental Health

CA + DM+ patients did not report worse mental health or emotional functioning compared to CA + DM- or CA-DM+ in 3 cross-sectional [21, 22, 27] and 1 longitudinal study [25]. One study included a measure of problems with anxiety, but no significant differences were found between prostate or breast CA + DM+ as compared to CA-DM+ patients in unadjusted analyses [23].

### Diabetes self-management

Two studies, using the same longitudinal data, addressed problems with diabetes self-management among 43 patients with a solid tumor and type 1 or 2 diabetes [19, 20]. One study showed that patients reported higher scores on symptom burden and lower scores on diabetes self-management after 8 weeks on chemotherapy as compared to baseline (mean 32.57 ± 4.49 vs 25.43 ± 3.81 and 45.86 ± 2.65 vs 50.84 ± 2.47, respectively) [20]. In addition, a moderate impact of cancer on diabetes self-management was observed, which mainly affected the ability to exercise, blood sugar monitoring, and ability to eat and drink. Moreover, in qualitative assessments many individuals indicated that they prioritized cancer care instead of diabetes care [20]. The other study mainly focused on predictors of diabetes self-management [19]. This study showed that living arrangements, years with DM, the total number of medications, baseline DM self-management, DM self-efficacy, and baseline and 8-week symptom severity were significant predictors of diabetes self-management, while diabetes complications, cancer type, stage and treatment, outcome expectancies, and anxiety and depression were not [19].

## Discussion

The majority of the included studies in this systematic review (i.e., 8 out of 10 studies) addressed HRQoL, self-perceived health, functioning or symptoms, and two studies, based on the same data, assessed diabetes self-management. In all included studies, CA + DM+ patients reported worse outcomes, but in 1 longitudinal study among prostate cancer patients, differences disappeared after adjustments [25]. CA + DM+ patients mainly scored lower on general HRQoL [22, 26], physical functioning [21, 23, 27], and sexual functioning [27]. In addition, prostate CA + DM+ patients reported lower urinary functioning [24] and lower vitality [22], while colorectal CA + DM+ vs CA + DM- patients reported more neuropathic symptoms in a cross-sectional study [28]. Finally, among diabetes patients that also had concurrent cancer, symptom severity increased and diabetes self-management, mainly exercise, blood sugar monitoring, and the ability to eat and drink, was impaired after 8 weeks on chemotherapy [20].

Similar to the results found in our systematic review, literature shows that comorbidity has a significant impact on HRQoL. Several other studies that were not included in this review but included diabetes as one of the studied comorbid conditions showed that cancer patients with comorbidity reported lower HRQoL or functioning [30–33]. A few of those studies reported the impact of diabetes separately and found a poorer general health [30], lower physical functioning [30, 33], more symptoms of nausea [31], and more erection problems among CA + DM+ men [32]. In line with these results, the number of comorbidities, including cancer, among patients with diabetes has also been shown to result in poorer HRQoL [34]. These studies were excluded from the present review as CA + DM+ patients were not the main sample, and as a result the number of included patients with both diseases was often low.

Although the included studies were of adequate to high quality, they differed substantially in design, population, and methodology. Different instruments were used to measure HRQoL which hampers comparison of the results. Moreover, different cancer types were studied and sample sizes in subgroups were generally low, particularly for CA + DM+ patients. The majority of studies included CA + DM+ and CA + DM- patients, although some studies additionally included a normative sample or CA-DM+ patients for comparison. As a result, information regarding diabetes characteristics was scarce with only 3 out of 10 studies including clinical data regarding diabetes. However, it is important to take the duration and severity of diabetes into account as this may influence the outcomes. Only 4 prospective studies were included, of which 2 were based on the same data, and these studies were conducted mainly among prostate cancer patients.

Despite the heterogeneity in patient samples and PROs studied, this systematic review also has several strengths. It is the first to summarize the literature on PROs among CA + DM+ patients. In addition, a broad search strategy was used and thereby a complete overview of the previous literature is presented. Finally, the quality of all included studies was assessed by two independent investigators with a 14-item checklist.

### Directions for future research

Although previous studies suggest that having both cancer and diabetes results in worse outcomes, the evidence is scarce and many relevant topics have not been studied yet. This systematic review shows that the majority of studies focused on general HRQoL and physical function, however, only little attention has been paid to mental health. Mental health was assessed in 5 of the 10 included studies but did not appear to be deteriorated in CA + DM+ patients as compared with CA + DM- and CA-DM+ patients. However, this might be a result of the used instrument, as all studies used a subscale of a HRQoL instrument, which might not be sensitive to more specific symptoms of anxiety or depression. Depression is a common problem in both cancer and diabetes patients. Previous research shows that depression is highly prevalent, in about a third of all cancer as well as diabetes patients and is associated with worse prognostic outcomes [35–38]. Therefore, it is possible that CA + DM+ patients might encounter more mental health issues, which were not picked up in the limited studies in this review. Thus, future studies should focus on mental health issues, including depression among CA + DM+ patients.

Previous studies show that among both cancer and diabetes patients BMI, physical activity, and smoking are significant predictors of HRQoL [39–43]. However, only 4 of the studies included in this review adjusted for lifestyle factors of which 3 only included BMI [24, 27, 28] and 1 study additionally adjusted for physical activity and smoking [26]. These studies showed that CA + DM+ patients have a higher BMI [24, 26–28] and are less physically active [26] at baseline than those without diabetes. Although, these studies did observe lower HRQoL among CA + DM+ vs CA + DM- patients independent of the adjustment for lifestyle factors, more research is needed. It is important to assess whether the poorer lifestyle, rather than clinical factors, of CA + DM+ patients is responsible for the lower HRQoL in this group. Moreover, future research should focus on the effect of changes in lifestyle factors and their impact on HRQoL; with that knowledge, interventions can be developed to improve HRQoL on the long term.

Elderly often live with several chronic illnesses such as cancer and diabetes, which poses a burden on patients. Due to the improved survival, self-management of these chronic diseases is becoming more important. This review included two studies on diabetes self-management which showed that cancer patients performed fewer diabetes self-management behaviors, such as monitoring of the blood glucose levels and exercising, after 8 weeks on chemotherapy [20]. Moreover, qualitative research showed that diabetes patients who develop cancer prioritize their cancer care over their diabetes care [20]. Among diabetes patients, self-management is widely studied and a previous literature review and meta-analysis shows that self-management interventions can improve blood glucose levels, increase knowledge and self-efficacy, and eventually might reduce costs of healthcare utilization [44]. It is important that both patients as well as specialists recognize the importance of self-management of multiple chronic illnesses. It is important that patients are able to utilize their resources and feel that they are in control of life and solve problems when necessary. Therefore, we believe that empowerment of patients and improving self-management behavior are important topics to address in future studies among patients with multiple chronic diseases.

## Conclusion

In conclusion, this systematic review indicates that having both cancer and diabetes results in worse PROs. However, a relatively low number of studies were included and no definitive conclusions can be drawn because of the heterogeneity of the included studies. The included studies were of reasonable quality but a main issue was that clinical information regarding diabetes was missing. More prospective studies with sufficient sample sizes are needed to establish these findings. As this research area is largely neglected and the majority of studies focused on HRQoL and physical function, future research should focus on other PROs that are highly prevalent among both cancer and diabetes patients such as mental health, including depression. In addition, as the occurrence of multiple chronic diseases poses important constraints on a person’s life and their health care, topics such as self-care and patient empowerment should receive more attention in future research.

## Electronic supplementary material

ESM 1 (DOCX 26 kb)
