# Supplementary material for: The impact of having both cancer and diabetes on patient-reported outcomes: a systematic review and directions for future research
Source: J Cancer Surviv. 2015 Oct 1;10:406–15. doi: 10.1007/s11764-015-0486-3 (PMC4801990; doi:10.1007/s11764-015-0486-3)
Supplement: Supplementary file 1 — (DOCX 26 kb) [file 11764_2015_486_JMOESM1_ESM.docx]

**Online Resource 1**

**Article Title:** The impact of having both cancer and diabetes on patient reported outcomes: a systematic review and directions for future research **Journal name:** Journal of Cancer Survivorship
**Author names and affiliations:** Pauline A.J. Vissers^1,2^, Louise Falzon^3^, Lonneke V. van de Poll-Franse^1,2^, Frans Pouwer^1^, Melissa S.Y. Thong^1,2^

^1^ C*o*RPS - Center of Research on Psychology in Somatic diseases, Department of Medical and Clinical Psychology, Tilburg University, The Netherlands
^2^ Department of Research, Netherlands Comprehensive Cancer Organisation, Eindhoven, The Netherlands
^3^ Center for Behavioral Cardiovascular Health, Columbia University Medical Center, New York, USA
**Email address corresponding author:** p.a.j.vissers@tilburguniversity.edu

**All databases were searched on August 28, 2013 and updated on March 31, 2014 and on January 27, 2015**

**MEDLINE (Ovid and Ovid MEDLINE(R) In-Process & Other Non-Indexed Citations)**1. exp *Diabetes Mellitus/
2. diabet$.ti.
3. 1 or 2

4. exp *Neoplasms/

5. (cancer$ or neoplasm$ or oncolog$).ti.

6. 4 or 5

7. 3 and 6

8. randomized controlled trial.pt.

9. controlled clinical trial.pt.

10. randomized.ab.

11. placebo.ab.

12. drug therapy.fs.

13. randomly.ab.

14. trial.ab.

15. groups.ab.

16. or/8-15

17. (animals not (humans and animals)).sh.

18. 16 not 17

19. 7 and 18

20. exp Epidemiologic Studies/

21. cohort$.tw.

22. (case$ and control$).tw.

23. (case$ and series).tw.

24. case reports.pt.

25. (case$ adj2 report$).tw.

26. (case$ adj2 stud$).tw.

27. Cross-Sectional.tw.

28. prevalen$.tw.

29. retrospective.tw.

30. or/20-29

31. (animals not (humans and animals)).sh.

32. 30 not 31

33. 7 and 32

34. 19 or 33

35. limit 34 to english language

**The Cochrane Library**#1 MeSH descriptor: [Diabetes Mellitus] explode all trees

#2 diabet*:ti

#3 #1 or #2

#4 MeSH descriptor: [Neoplasms] explode all trees

#5 (cancer* or neoplasm* or oncolog*):ti

#6 #4 or #5

#7 #3 and #6

**CINAHL (EBSCOhost)**

S1 (MM "Diabetes Mellitus+")

S2 TI diabet*

S3 S1 OR S2

S4 (MM "Neoplasms+")

S5 TI ( cancer* or neoplasm* or oncolog*)

S6 S4 OR S5

S7 S3 AND S6

**PsycINFO (Ovid)**

1. diabetes mellitus/

2. diabet$.ti.

3. 1 or 2

4. exp neoplasms/

5. (cancer$ or neoplasm$ or oncolog$).ti.

6. 4 or 5

7. 3 and 6
